# Electric Scooter‐Related Deaths in Queensland, Australia

**DOI:** 10.1111/ans.70915

**Published:** 2026-08-27

**Authors:** Nathan Milne, Jack Garland, Rexson Tse, Beng Beng Ong

**Affiliations:** ^1^ Forensic Pathology and Coronial Services Brisbane Australia; ^2^ School of Medicine, The University of Queensland Brisbane Australia; ^3^ Faculty of Health Sciences and Medicine, Bond University Gold Coast Australia; ^4^ School of Medicine and Dentistry, Griffith University Gold Coast Australia; ^5^ Forensic Pathology and Coronial Services Gold Coast Australia

**Keywords:** accidents, cause of death, electric scooter, e‐scooter, forensic pathology, wounds and injuries

## Abstract

**Background:**

The use of electric scooters (e‐scooters) has become an increasingly common means of transport in various parts of the world, as an affordable form of short distance transport. However, there are associated problems related to limitations of pre‐existing infrastructure, legislative considerations, and injuries and deaths resulting from their use. There have been numerous studies regarding non‐fatal e‐scooter injuries, but few studies of fatalities.

**Methods:**

This paper reviews all e‐scooter‐related deaths in the state of Queensland, Australia, including deaths resulting from e‐scooter battery fires.

**Results:**

Twenty‐two deaths were recorded between 2019 and October 2025, with 19 resulting from collisions while being ridden, and three resulting from battery fires while they were charging. All collision‐related deaths were of single riders with 16/19 having at least one identified factor as a potential contributor to the accident, including excessive speed, alcohol intoxication and failure to give way. Head injuries were the most common cause of death, and of note there was poor compliance with the legally mandated use of helmets, with 13/19 either not wearing a helmet or wearing one incorrectly.

**Conclusion:**

Deaths from e‐scooters increased between 2019 and 2024 and were usually the result of head injuries in the context of poor helmet compliance and other behavioural characteristics. Further research comparing e‐scooter deaths with other transport modalities is recommended.

## Graphical abstract

E‐scooter usage has risen in recent years, and Queensland has seen increasing numbers of e‐scooter‐related deaths. Potential factors associated with these fatal collisions include excessive speed, alcohol intoxication, failure to give way and poor compliance with helmet use.

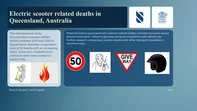

## 1 Introduction

Electric scooters (e‐scooters) have emerged in recent years as an alternative means of transport. Their benefits include affordability, short distance travel, ease of use and parking and reduction of road congestion, pollution and noise [1, 2]. However, these have come with associated problems such as irresponsible or dangerous riding, cluttering of pedestrian spaces, competition for pedestrian, bicycle and car paths and roads, and adding complexity to transport systems that do not have existing infrastructure to accommodate e‐scooters [1, 2]. This creates regulatory and legislative challenges, including safety concerns for both the rider and other path and road users, as e‐scooter accidents can lead to serious injury and death. It has been reported in e‐scooter websites that the fastest e‐scooters are capable of reaching speeds of 160.9 km/h (100 mph) [3] and can accelerate from stationary to 24.1 km/h (15 mph) in as little as 3.8 s [4].

Queensland is the third most populous state of Australia, with a decentralised population of approximately five million people. A shared e‐scooter scheme was first introduced to the capital city, Brisbane, in November 2018 [5]. Despite the negative aspects of e‐scooters, the Queensland Government Strategy of 2020 states that further transport initiatives to investigate include the provision of more shared services such as e‐scooters [6]. E‐scooters are also available for private ownership. Legislation relating to e‐scooters varies between the different states and territories of Australia. The current rules for riding an e‐scooter in Queensland have been in place since November 2022. The rider does not need to have a licence, and the e‐scooter does not need to be registered. The rules include being at least 16 years old or at least 12 years old under supervision, a speed limit of 12 km/h on footpaths and shared paths and 25 km/h on separated and bicycle paths, no ‘doubling’ of another person, no intoxication by alcohol or drugs and a bicycle or motorcycle helmet must be worn [7]. Although helmets are provided with hire e‐scooters, their availability has been complicated by theft, with an estimated 11 000 helmets stolen in the first 6 months of the hire scheme [8]. An observational study of e‐scooter riders in Brisbane showed that 45% were being ridden illegally, and helmets were being correctly worn by only 60.9% of riders of shared e‐scooters and 95% of private e‐scooters [9]. A radiological study of e‐scooter injuries in Brisbane noted that the use of a helmet was only sparsely recorded in emergency department notes [10].

E‐scooter injuries have been reported in numerous countries [11, 12, 13, 14, 15, 16] and several Australian jurisdictions [10, 17, 18, 19, 20, 21]. The frequency of injuries requiring hospital presentation has imposed a significant burden on the health system. For example, a major hospital in Brisbane had 54 e‐scooter‐related injury presentations over a 2‐month period [18], and 443 presentations over a 12‐month period [10], with the latter study indicating more than 1400 radiological investigations were required.

However, there are few studies examining e‐scooter‐related fatalities in the literature. An American study of 21 deaths showed that almost all were male and the driver of the e‐scooter, most deaths occurred at night and were the result of a collision with a motor vehicle and adverse environmental conditions were commonly present [22]. A Polish study of nine deaths also showed a male predominance, and also noted rare helmet use and deaths commonly occurring on weekdays during the morning rush hour [23].

This study examines all e‐scooter‐related deaths that have occurred in Queensland, including those that have resulted from e‐scooter battery fires while it was charging. It examines the lethal injuries and circumstances surrounding the death, with the aim of determining common characteristics present in fatalities. The identification of such common characteristics may guide future research or legislative changes related to e‐scooter usage.

## 2 Methods

### 2.1 Case Selection

This study was a review of all e‐scooter‐related deaths since their introduction into Queensland.

The cases were identified using the ‘*Auslab*’ pathology database which records all Coronial autopsy reports dating back approximately 22 years. This encompasses all e‐scooter‐related deaths as all traumatic deaths are reportable under the Queensland Coroners Act 2003. The keywords ‘scooter’, ‘electric scooter’ and ‘e‐scooter’ were used to search autopsy reports. Cases were included in the study if the death was directly related to e‐scooters. This included deaths from e‐scooter battery fires, not just incidents whilst operating/riding an e‐scooter. Further information was obtained from Coronial findings, which include additional information and circumstances and conclusions that are not available in the autopsy reports. Coroners' findings were available for 13/22 cases. Queensland Fire and Rescue Service reports and Forensic Crash Unit reports were also reviewed, where available.

The details obtained from these cases, if available, were the sex, age, time of day, day of the week, cause of death, lethal injuries, toxicology results, mechanism of the incident, whether a helmet was worn, if the deceased was the driver, if the e‐scooter was hired or private, the duration of any survival period and the presence of any potentially contributory natural disease. Death types were divided into collision deaths and battery fire deaths. The term collision encompasses both direct impacts and falls from an e‐scooter. The reason for this grouping is that the two mechanisms frequently overlap in the same incident, and collisions broadly involve the impact of an e‐scooter with any other object including other moving vehicles and stationary objects (such as parked vehicles, but also fixed objects such as walls and protrusions of a pathway/road).

### 2.2 Exclusion Criteria

All cases of e‐scooter‐related deaths were included in the study.

### 2.3 Statistical Analysis

Statistical analysis was performed using packages in R (open source, R studio 2022.07.01, Build 554, R version 4.2.2) and a *p*‐value of < 0.05 was considered significant. Summary and descriptive/proportion statistics are presented. The trend is explored using Spearman's correlation test. Fisher's exact test is used to explore any differences in categorical variables. Mann–Whitney test is used to explore any differences in continuous variables due to non‐parametric distribution (Shapiro–Wilks test).

### 2.4 Ethics Approval

Ethics approval for this study was granted by the Ethics Committee, Coronial and Public Health Sciences, Queensland Health.

## 3 Results

### 3.1 Overall Deaths and Trend

In the study period, there were 22 e‐scooter‐related deaths, commencing in 2019. All cases underwent full‐body postmortem CT scan, with a combination of external only, partial internal and full internal autopsy examinations. There was one death in 2019, one in 2020, one in 2021, four in 2022, three in 2023, seven in 2024 and five in 2025 (thus far at time of study), showing an increase between 2019 and 2024 with a significant Spearman's correlation (*ρ* = 0.88, *p* < 0.05).

### 3.2 Demographics and Medical Disease

Figure 1 outlines the age demographic data. There was a higher proportion of male (M:F = 19:3). The mean age was 40 years (median: 35.5, standard deviation: 18.4, min: 12, max: 75), with 15 cases being less than 50 years old. There was some degree of hospital survival in 11/22 cases, ranging from 1 to 21 days, with 1 day (3/11) and 4 days (3/11) being the most common. Some element of background cardiac disease (two cases of coronary atherosclerosis and one case of coronary atherosclerosis, cardiomegaly and atrial fibrillation) was noted in 3/22 cases, but only listed as a significant condition contributing to death in one case.

**FIGURE 1:**
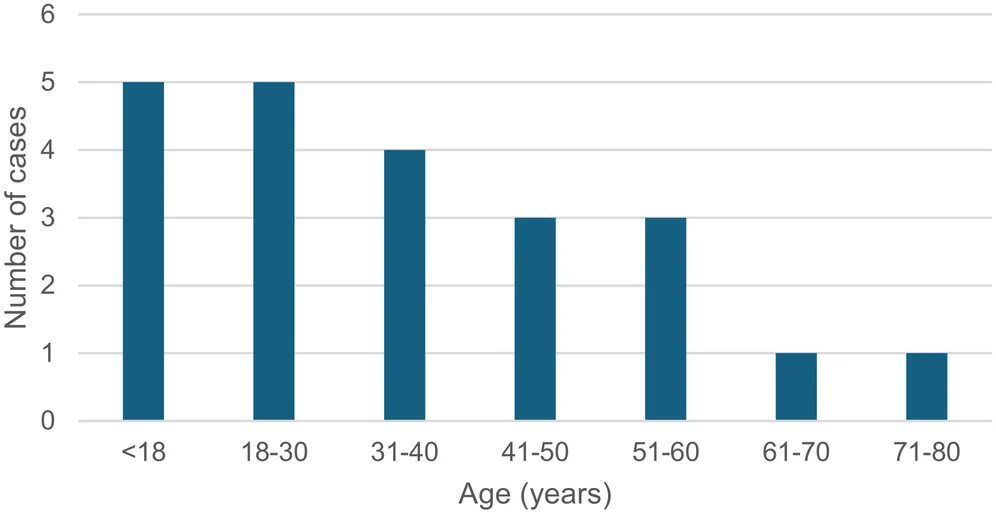
Age demographics.

### 3.3 Type of Scooter

For 2/22 cases the private‐versus‐hire status of the scooter was not known, for 17/22 cases (85% of cases with known private‐vs.‐hire status) involved a private e‐scooter, and 3/22 cases (15% of cases with known private‐vs.‐hire status) were hired e‐scooters. Nineteen deaths were collision‐related (all of whom were the e‐scooter rider/not‐pedestrian, and were solo riders) and three deaths were the result of e‐scooter battery fires (three separate incidents where the deceased was the e‐scooter owner in all cases).

### 3.4 Day and Time

All days of the week were represented in collision deaths, with Tuesday and Wednesday the most common (five cases each) and Monday, Thursday and Sunday the least common (one case each). Figure 2 outlines the day of week collision data. Of the collision deaths 10/19 occurred at night, 1/19 occurred at twilight and 8/19 occurred during daylight. Only 1/19 occurred on a weekday morning during the busy commuting period. Due to overall low number of cases, no comparative statistics were performed.

**FIGURE 2:**
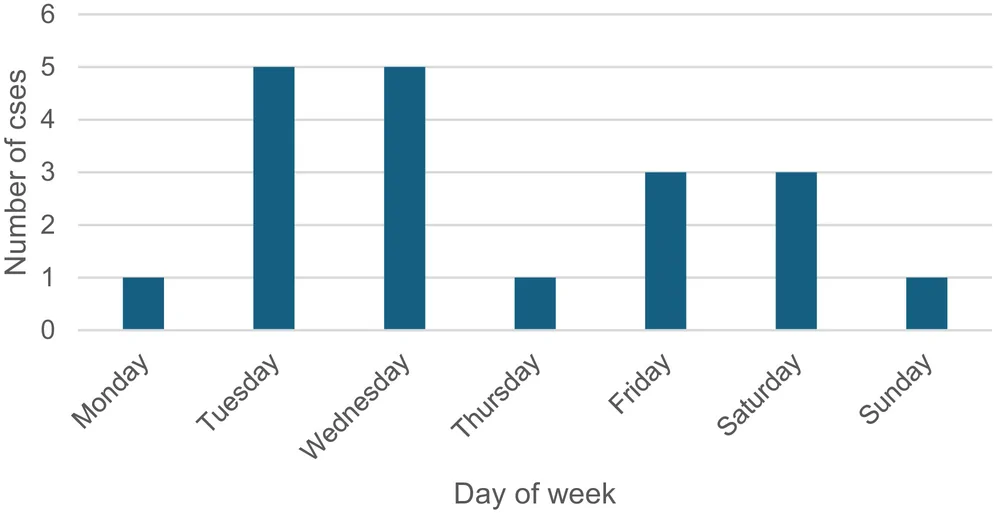
Day of week of collision.

### 3.5 Mechanism of Death

Of the three fire‐related deaths, all occurred at home whilst the battery was charging. Two of these three cases were the result of the wrong charger type for the battery. In the remaining case, the fire occurred as a result of thermal runaway of undetermined cause (causes can include physical damage, overheating and failure of the battery management system). Carbon monoxide was present at levels of 3%, 32% and 47% in the fire‐related deaths. Only the low (3%) carbon monoxide level death was associated with any survival period (1 day in hospital). This case demonstrated 90% total body surface area burns, which was the cause of death. The case with the 47% carbon monoxide level showed 70% total body surface area burns and the cause of death was effects of fire (therefore inclusive of burns and carbon monoxide effects). The case with the carbon monoxide level of 32% showed no injuries and the cause of death was given as smoke inhalation.

Of the collision deaths, the mechanism of death was traumatic in 17/19 cases, and due to positional asphyxia in 2/19 cases. Six of 19 collision deaths involved a road collision with a moving motor vehicle (car or truck). Of the six deaths involving collision with a moving motor vehicle, the motor vehicle was found to be primarily at fault in 1/6 cases (the motor vehicle was speeding), the e‐scooter rider was found to be primarily at fault in 3/6 cases and in 2/6 cases investigation findings were not yet available. Of the traumatic deaths, the sites which involved lethal injury unsurprisingly included the head in 14/17 cases (9/17 of which were head‐alone, with the next most‐common co‐present site being the chest). Of the three non‐head lethal injury sites, 1/17 involved the neck, 1/17 involved the chest and 1/17 involved the chest, abdomen and pelvis.

### 3.6 Toxicology

In terms of toxicological analyses, alcohol was present above 0.05% (legal limit for driving or operating an e‐scooter in Queensland, Australia) in the 6/7 cases where it was detected (ranging from 0.034%, which was in a fire death, up to 0.27%). All six cases where alcohol was above 0.05% were riders of scooters at the time of collision. Of the cases where non‐toxic levels of drugs or illicit drugs were detected (7/22 cases), 3/7 involved methylamphetamine (one of which was a fire death), 1/7 involved cocaine metabolites, 1/7 involved a potentially toxic range level of sertraline and 2/7 detected cannabis. In 2/22 cases testing was not possible due to prolonged hospital admission with discarded blood samples from time of admission.

### 3.7 User‐Related Contributing Factors

Figure 3 outlines the user‐related potential contributing factors to collision in the collision deaths. Sixteen of 19 collision deaths had user‐related potential contributing factors to collision, which included a variety of overlapping characteristics, of which the most common was excessive speed (9/19 cases, all of which involved private e‐scooters therefore capable of being modified to exceed legal speed restrictions of 25 km/h maximum on public roads or paths), followed by the aforementioned alcohol level of > 0.05% in 6/19 cases (an alcohol level below the legal limit was not attributed as being an impairment to operation of an e‐scooter), failure to give way (3/19 cases), and defective brakes on a private e‐scooter (3/19). There was no significant difference (Fisher's exact test, *p* > 0.05) between younger (< 50 years old) and older (> 50 years old) deaths in proportion of operating in excessive speed, presence of alcohol and failure to give way.

**FIGURE 3:**
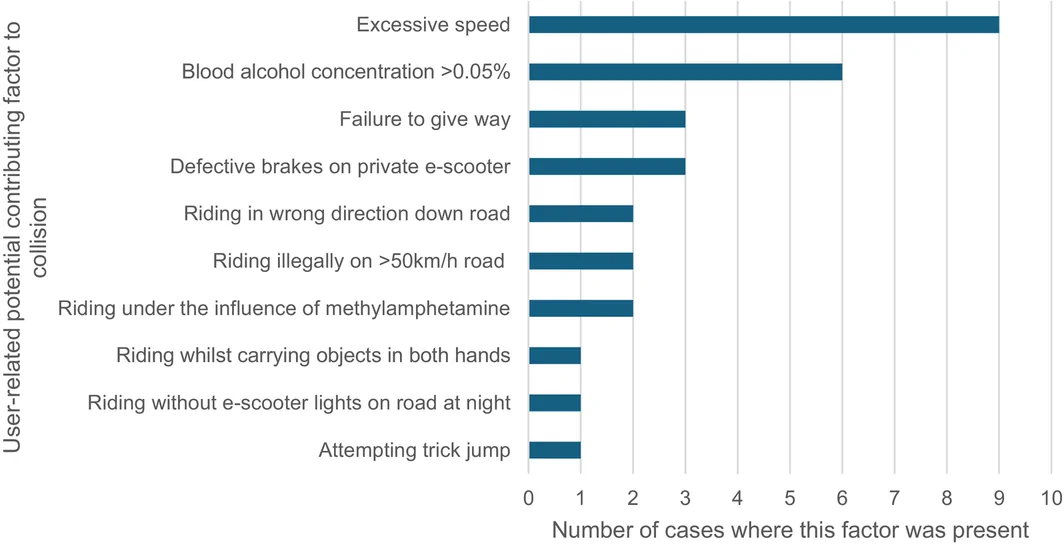
User‐related potential contributing factors to collision.

Regarding non‐alcohol drugs detected on toxicology, only methylamphetamine was considered to have a potential attributable impairment on operation of an e‐scooter out of the detected drugs. This is because methylamphetamine has a wide toxic range and its presence indicates current physiological effects (in contrast to cocaine metabolites and cannabis). Cocaine metabolites and cannabis were not included (as their presence may represent prior use with no current major physiological effects), and the sertraline level being in a potentially toxic range was not considered to be a sufficient physiological contributor to collision (particularly as it is prone to postmortem redistribution and may be artefactually elevated).

Incorrect use of or lack of helmet was recorded as a potential user‐related contributing factor for fatal outcome of collision. Of the 19 collision deaths, 6/19 were wearing helmets, 12/19 were not wearing helmets and 1/19 was wearing the helmet incorrectly (backwards). Therefore, in 68.4% of cases of fatal e‐scooter collision, the rider was either not wearing a helmet, or not wearing the helmet correctly. Of the 13 deaths who were either not wearing helmet or wearing helmet incorrectly, 12 of them had documented head injuries (> 90%). This was proportionally higher than those six deaths who wore helmets correctly with only two (33.3%) having documented head injuries but did not reach statistical significance (Fisher's exact, *p* > 0.05). Younger individuals (< 50 years old) had 11 deaths with incorrect or not wearing helmets, and two wearing it properly. This was proportionally higher compared with older individuals where there were two deaths with incorrect or not wearing helmets, and four wearing it correctly, but did not reach statistical significance (Fisher's exact, *p* > 0.05).

Combining the user‐related potential contributing factors to collision with the helmet non‐compliance data, 18/19 collision deaths had either a user‐related potential contributing factor to collision or helmet non‐compliance. Thirteen of 19 cases had more than one user‐related potential contributing factor to collision or helmet non‐compliance. In the 1/19 collision‐related death where there was no user‐related potential contributing factor to collision or helmet non‐compliance, the Coronial inquest found that the collision occurred as a result of a pothole in the sidewalk compounded by poor lighting at 5 AM. Younger individuals (< 50 years old) had an average of 2.2 user‐related potential contributing factors which was significantly higher than in the older individuals with 1.1 contributing factors (Mann–Whitney *U*‐test, *p* < 0.05).

## 4 Discussion and Limitations

The results demonstrated a number of expected and unexpected findings. Expectedly, head injuries were over‐represented (present in 82% of traumatic e‐scooter deaths) in the cohort of fatal e‐scooter collisions. Similarly, poor helmet compliance (helmet non‐compliance in 68.4% of fatal collisions) was also unsurprising given the cohort was composed of fatal e‐scooter incidents with a high proportion of head injuries. This is consistent with previous studies on bicycle crash data showing that helmet use is associated with reduced severity of head injury [24]. For the cases with poor helmet compliance, 12/13 (92.3%) sustained head injuries causing death, either in isolation (8/12) or in combination with injuries to other regions of the body (4/12). Given that hire scooters are speed‐limited in Queensland, the over‐representation of private scooters in fatal e‐scooter collisions was not unexpected, particularly as excessive speed was noted in nearly half of the collisions.

Somewhat unexpectedly, all e‐scooter collision deaths in Queensland exclusively involved a sole rider, with no associated pedestrian or passenger deaths. Given that e‐scooters are legal to use in Queensland on bike paths, shared pedestrian paths, and roads, this is in great contrast to motor vehicle collisions which frequently involve the deaths of passengers and other parties. Australian motor vehicle deaths involve passengers one‐third to one‐half as often as drivers [25, 26], and pedestrians comprise approximately 14% of all road fatalities [27, 28]. Comparing to published pedal cyclist death data, the majority of cyclist deaths also involve head injuries; however, are most commonly due to collision with a motor vehicle and are usually compliant with helmet use [26].

When comparing the results with two other multiple fatality studies [22, 23], similarities included the majority of deaths being male riders, and there being user‐related factors such as poor helmet compliance, alcohol intoxication and illegal operation of the e‐scooter contributing to the event. Our study had a lower proportion of collisions with motor vehicles (6/19) compared with the American study which included deaths from 15 cities (18/21) [22]. Of interest, the Polish study noted 4/9 deaths occurred on weekdays between 7:00 and 10:30 AM, while our study had only 1/19 in the same period [23]. A possible explanation for this could be regional differences in using e‐scooters for transportation or recreational purposes.

There are several important limitations to this study. Firstly, the overall numbers of e‐scooter deaths are quite low, with pedestrian road traffic deaths alone in 2024 (the highest year of e‐scooter deaths in Queensland so far) being over triple that of e‐scooters [29]. Whilst this may mean that e‐scooters are relatively safe, at least from a fatality perspective, it is difficult to make comparisons without an understanding of the per‐population hours of e‐scooter riding compared with pedestrians or other modes of transportation. The overall numbers of e‐scooter fatalities are relatively low but increasing, and therefore caution is recommended if attempting to extrapolate historical fatal collision attributes to future events. Another limitation is the inclusion of only fatal e‐scooter cases (a necessity inherent to forensic pathology data). Hospital admission statistics for e‐scooter injuries far exceed fatal outcomes and inferences cannot be drawn about e‐scooter injuries more broadly from a fatality dataset that will be skewed towards extreme injuries. Data on even severe non‐fatal e‐scooter collision outcomes (including life‐long disability) are not included in this study's dataset.

## 5 Conclusions

E‐scooters continue to increase in popularity as local laws struggle to keep up with evolving access to ownership (and modification) of these devices. As an increasingly common form of transportation that was almost unheard of 10 years ago, there is increased public and political focus on potential dangers associated with these devices. Much of the published literature on this topic deals with non‐fatal injury data, and there is limited published data from a forensic pathology fatality‐focused perspective. This study reviewed all e‐scooter deaths recorded in the State of Queensland, Australia. Of the 22 deaths, 19 were collision related and three were battery‐fire related. The current data suggest excessive speed and helmet non‐compliance to be over‐represented in e‐scooter deaths. For battery‐fire deaths, two of the three cases were confirmed to be the result of use of the wrong charger for the battery type. Future per‐population comparison with other modes of transportation is recommended.

## Author Contributions

**Nathan Milne:** conceptualisation, methodology, writing – original draft, writing – review and editing, project administration, investigation. **Rexson Tse:** conceptualisation, methodology, investigation, formal analysis, writing – original draft, writing – review and editing. **Beng Beng Ong:** conceptualisation, methodology, investigation, writing – original draft, writing – review and editing. **Jack Garland:** conceptualisation, methodology, investigation, visualisation, writing – original draft, writing – review and editing.

## Conflicts of Interest

The authors declare no conflicts of interest.

## Data Availability

Research data are not shared.
